# The Neural Network of Neuropeptide S (NPS): Implications in Food Intake and Gastrointestinal Functions

**DOI:** 10.3390/ph14040293

**Published:** 2021-03-26

**Authors:** Luca Botticelli, Emanuela Micioni Di Bonaventura, Massimo Ubaldi, Roberto Ciccocioppo, Carlo Cifani, Maria Vittoria Micioni Di Bonaventura

**Affiliations:** School of Pharmacy, University of Camerino, via Madonna delle Carceri, 9, 62032 Camerino, Italy; luca.botticelli@unicam.it (L.B.); emanuela.micioni@unicam.it (E.M.D.B.); massimo.ubaldi@unicam.it (M.U.); roberto.ciccocioppo@unicam.it (R.C.); mariavittoria.micioni@unicam.it (M.V.M.D.B.)

**Keywords:** Neuropeptide S (NPS), NPS receptor (NPSR), central nervous system, food intake, anorexigenic peptide, CRF, Orexin-A, gastrointestinal function, eating behavior, stress

## Abstract

The Neuropeptide S (NPS), a 20 amino acids peptide, is recognized as the endogenous ligand of a previously orphan G protein-coupled receptor, now termed NPS receptor (NPSR). The limited distribution of the NPS-expressing neurons in few regions of the brainstem is in contrast with the extensive expression of NPSR in the rodent central nervous system, suggesting the involvement of this receptor in several brain functions. In particular, NPS promotes locomotor activity, behavioral arousal, wakefulness, and unexpectedly, at the same time, it exerts anxiolytic-like properties. Intriguingly, the NPS system is implicated in the rewarding properties of drugs of abuse and in the regulation of food intake. Here, we focus on the anorexigenic effect of NPS, centrally injected in different brain areas, in both sated and fasted animals, fed with standard or palatable food, and, in addition, on its influence in the gastrointestinal tract. Further investigations, regarding the role of the NPS/NPSR system and its potential interaction with other neurotransmitters could be useful to understand the mechanisms underlying its action and to develop novel pharmacological tools for the treatment of aberrant feeding patterns and obesity.

## 1. Introduction

Obesity is a worldwide health problem together with its related comorbidities, such as type-2 diabetes, cardiovascular diseases, or dyslipidemia, that severely reduces the quality of life [1,2]. The understanding of the biological mechanisms, that control food intake and energy homeostasis, is of a primary importance for the discovery of novel potential pharmacological tools for the treatment of this disease [1,2]. In the brain, despite the large number of regions implicated in eating behavior, the hypothalamus is considered as the main feeding center for the control of appetite, in which a great number of neuropeptides is involved in the modulation of food intake and body weight [3,4,5]. The interaction among these peptides in the central nervous system (CNS) results in a complex regulation of food intake and, the dysregulation of their neurotransmission can contribute to increased appetite, weight gain, and consequently, obesity [6]. Among them, the neuropeptide Y (NPY), Agouti-related-peptide (AgRP), melanin-concentrating hormone (MCH), or orexins are some of the most potent stimulators of food intake [7,8,9,10], while others, such as melanocortins, oxytocin (OXT), corticotropin-releasing factor (CRF), or cocaine- and amphetamine- regulated transcript show anorexigenic effects [11,12,13,14].

Among the anorexigenic circuitries in the CNS, the neuropeptide S (NPS) system gained more and more interest in the recent years. The first investigation of the behavioral effects induced by the NPS demonstrated that it was able to promote wakefulness, locomotor activity, arousal and to exert anxiolytic-like effects in rodents [15], but interestingly, several following studies highlighted the potent inhibitory effect of NPS on food intake, in different animal species [16,17,18,19]. Moreover, in humans, polymorphisms of the NPSR gene were recently associated with an increased susceptibility to obesity, and NPS serum levels were found lower in individuals with a high body mass index [20]. The influence of NPS on food intake is consistent with the presence of the NPS receptor (NPSR) in feeding-related brain areas, such as the Arcuate Nucleus of the hypothalamus (ARC), lateral hypothalamic area, paraventricular nucleus of the hypothalamus (PVN) and the dorsomedial hypothalamic nucleus (DMN) [15,21], and with the interconnection between NPS and the other neurotransmitters implicated in feeding behavior.

In this review, we will first describe the most important biological, neuroanatomical, and pharmacological features regarding the NPS system, and then, we will focus on the role played by the NPS and its receptor on food consumption, revising the current literature linking this system to feeding behavior, considering that it could represent an important pharmacological target to treat obesity and eating disorders.

## 2. The NPS System

The NPS, an example of neurotransmitter discovered with a reverse pharmacological approach, is a linear 20 amino acids peptide, identified as the endogenous ligand of the previously orphan G protein-coupled receptor (GPCR) GPR154 [15,22], now recognized as the NPSR. The primary structure of the NPS, which shares no homology with the other neuropeptides, is highly conserved among vertebrates, whereas the gene appears absent in DNA sequences of fish, amphibian, and reptile, presumably indicating the NPS as a relatively recent gene during vertebrate evolution and that it is specifically present in tetrapods [23]. The name “Neuropeptide S” was proposed considering that serine is the amino-terminal residue found in the primary structure of the mature peptide among all different species [15,24,25].

Originally known as GPR154, or G protein-coupled receptor associated with asthma (GPRA), NPSR was identified through de-orphanization studies and has two main isoforms: the shorter NPSR1-A and longer NPSR1-B [26]. The NPSR is a typical GPCR, characterized by seven membrane-spanning domains and it shares moderate homology with the other members of the supergene family. In particular, the highest levels of similarity were found with the vasopressin and OXT receptors [15,24,25,27,28].

In vitro pharmacological studies, performed on cell lines stably expressing human and murine NPSRs, revealed that NPS acts as an excitatory neurotransmitter, in light of the ability to elevate intracellular-free Ca^2+^ and to stimulate cAMP synthesis, at nanomolar concentrations and in a dose-dependent manner, indicating that the NPSR couples to both G_q_ and G_s_ protein, and thus increases cellular excitability [15,25,29,30]. Additionally, radioligand binding assays demonstrated that the radiolabeled NPS analog (^125^I-labeled Tyr^10^-NPS) shows a displaceable binding with increasing concentrations of NPS (Kd = 0.3 nm), suggesting that NPS behaves as a typical neurotransmitter, binding and activating its cognate receptor with high potency and specificity [15,28].

The human NPSR gene, located on chromosome 7p14-15, is characterized by multiple single nucleotide polymorphism (SNPs) and splice variants, with some of these SNPs associated to an increased susceptibility to asthma and higher levels of IgE, as determined by genetic linkage studies [31]. One of these SNPs is responsible for the amino acid exchange Asn-Ile at position 107 (Asn^107^Ile) of the mature protein (SNP591694 A > T; ref. SNP ID: rs324981), while a C-terminal splice variant was found overexpressed in human asthmatic airway tissues [31].

The Asn^107^Ile mutation results in a gain of NPSR function, considering that NPS activates the NPSR Ile^107^ with approximately 10-fold higher potency, compared to the NPSR Asn^107^, conversely to the alternatively spliced C-terminal variant, which does not influence pharmacological properties of the receptor protein [29]. Interestingly, the Asn^107^Ile SNP revealed not only to affect the in vitro properties of the NPSR, but also a gender-specific association of this genotype with panic disorder, mainly observed in male patients [32] and recently, a study performed in male obese Pakistani individuals, highlighted the presence of this SNP as a risk factor for the development of obesity [20].

### 2.1. The Localization of the NPS System in the CNS

**Figure 1:**
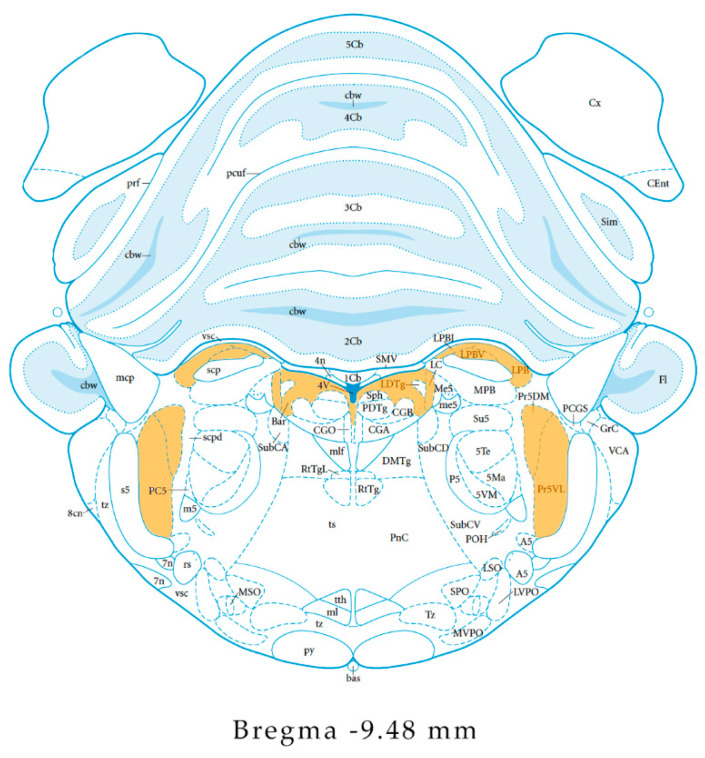
Localization of the Neuropeptide S (NPS)-producing neurons in the rat brainstem. The three principal regions of the brainstem reporting the expression of the NPS (the trigeminal principle sensory nucleus, the lateral parabrachial nucleus and the locus coeruleus area) are evidenced in orange. The drawing is adapted from the atlas of Paxinos & Watson (5th edition) [36].

In the rat brain, in situ hybridization studies revealed the presence of a group of NPS-expressing neurons in a region situated between the noradrenergic locus coeruleus (LC) and the Barrington’s nucleus, representing a previously unidentified population of neurons in this area of the brainstem which are not co-localized with either tyrosine-hydroxilase or CRF. In addition, NPS-neurons were also detected in the trigeminal principle sensory nucleus and they are co-expressed with vescicular glutamate transporter (VGLUT) mRNA, identifying these neurons predominantly glutamatergic, while only a small number of NPS-positive cells in the LC demonstrated to contain choline acetyltranferase (marker of cholinergic neurons). Strong expression of the NPS precursor mRNA has been additionally detected in the lateral parabrachial (LPB) nucleus, region in which was determined its co-expression with the CRF, presuming a possible interaction between NPS and excitatory and stimulatory neurotransmitters, such as glutamate, acetylcoline and CRF [15,21]. The three brainstem areas rich of NPS-expressing neurons are shown in Figure 1. Finally, expression of the NPS precursor has been observed in few scattered neurons of the amygdala and DMN [21].

The restricted distribution of the NPS precursor is in contrast to that of NPSR mRNA, which is widely expressed in the rat CNS, suggesting involvement of this receptor in a great number of brain functions. Using in situ hybridization studies, the highest levels of the NPSR mRNA were found in the cortex, thalamus, hypothalamus (particularly the PVN, DMN and ventromedial nucleus), parahippocampal formation, including the subiculum and amygdala, while less expression was detected in the brainstem, and no NPSR transcript was found in NPS precursor-expressing cells [15,21]. Subsequently, using a validated NPSR-specific antibody, Leonard et al. analyzed the expression of this protein in the rat brain, obtaining a consistent distribution pattern between the NPSR mRNA and protein [33].

The neuroanatomical distribution of the NPS system was also investigated in the mice brain, in order to elucidate the level of conservation between different rodent species. By in situ hybridization and immunohistochemistry approaches, Clark et al. found a strong expression of the NPS precursor only in the pericoerulear area and in the Kölliker-Fuse (KF) nucleus of the LPB area, while no signal was detected in the other areas of the brainstem [34].

A parallel study conducted by Liu et al., using transgenic NPS/EGFP mice, confirmed the previous findings, with NPS-producing cells confined in the same two nuclei of the brainstem [35], denoting a more limited expression of the NPS precursor in the mouse compared to rat brain. The same report also evidenced that NPS neurons in the pericoerulear area are predominantly glutamatergic and in the KF nucleus NPS was co-expressed with CRF, similarly to rat brain tissues [15,21,35].

In accordance with the distribution pattern documented in rats, mice showed a widespread expression of the NPSR mRNA throughout the brain, with higher levels detected in different regions of the cortex and, in particular, in subnuclei of the amygdala, suggesting the existence of species differences in certain brain areas, which could reflect divergent functions and organization of neuronal networks [34].

Figure 1 shows the localization of the NPS-expressing neurons in the rat brainstem.

### 2.2. Summary of the In Vivo Functions of NPS

The initial in vivo characterization of the NPS, performed in the study by Xu and collaborators, demonstrated that this neuropeptide, after intracerebroventricular (ICV) injections, was able to stimulate locomotor activity in both naive and habituated mice, suggesting that the NPS promotes behavioral arousal, independently of novelty or stress [15]. Since arousal is also a crucial component of wakefulness, it was investigated if NPS could influence sleep/wake patterns, and, accordingly, treatment with this neuropeptide suppressed all sleep stages in rats in the first hour post-administration [15]. Intriguingly, in light of NPSRs presence in the amygdala and hypothalamus, both implicated in emotional behaviors and stress responses [37,38,39], NPS administration attenuated behavioral signs of anxiety, as determined by four different paradigms (open field, light-dark box, elevated plus maze, and marble burying) [15]. Several studies, using different rodent paradigms and assays, replicated these results, highlighting the NPS unique behavioral profile in increasing locomotor activity, wakefulness, and paradoxically, at the same time, in exerting anxiolytic-like effects [18,40,41,42,43,44,45], in contrast to other neurotransmitters or psychostimulants drugs that promote arousal, such as cocaine or amphetamine that generally induce anxiety-like behaviors [15,24,25,28,46,47].

In addition, NPS demonstrated to reduce oxidative stress damage to lipids and proteins in the mouse brain [48,49], to promote antinociceptive effects at a supraspinal level [50,51] and to ameliorate spatial learning and memory impairment in rodents [52,53,54].

Finally, several studies suggested that the NPS system participates in the regulation of rewarding properties of drugs of abuse, such as morphine [55], cocaine [56,57], and ethanol [58,59], representing a potential target for the treatment of drug addiction (for review see [60]); moreover, the NPS might be also involved in obesity and aberrant eating behaviors, given the potent anorectic properties of this neuropeptide, as discussed in the next section.

## 3. NPS and Food Intake: Preclinical Studies

### 3.1. NPS in Rodents

NPS plays a role in regulating food consumption and eating behavior, predominantly showing an anorexigenic effect, capable to reduce appetite and counteract hyperphagia, even in the case of prolonged fasting. In Beck et al. study, the difference in standard chow intake was measured in male rats, that received an ICV injection of NPS or vehicle, after an overnight fast. Two doses of NPS were tested, 1 and 10 µg per rat: control rats ate significantly more than NPS-treated rats at both doses, which conversely, had a reduction of 55% of chow consumption in the first hour of refeeding. The highest dose revealed a longer lasting effect in decreasing food intake and, after 24 h, the rats ate significantly more quantity of food than the controls, presuming via compensatory mechanism; 48 h later all the animals ingested the same amount of chow [61]. Interestingly, levels of hormones, such as leptin, ghrelin, and insulin, known to be essential for the regulation of appetite [62,63,64], were not significantly modified by ICV injection of NPS.

In order to better understand the effect of this neuropeptide, the consumption of highly palatable food (HPF) in sated rats for 1 h was also evaluated, finding that the control rats ate almost the double of NPS rats injected with 10 µg, and, when refed on chow, both groups of rats consumed the same amount of standard food. The re-exposure to HPF 24 h after NPS injection revealed no difference in feeding between the groups of animals. In light of the elevated hypothalamic levels of the NPY in fasting state [65,66], the same study investigated a possible interaction with this orexigenic peptide [67,68,69], injected alone or co-administered with NPS, evidencing the incapacity of NPS to suppress food intake stimulated by NPY in sated rats, indicating an absence of interaction between these two neuropeptides. Thus, the anorectic action of NPS is probably due to other appetite-related mechanisms and is consistent with the expression of the NPSR in the DMN [15], brain region strictly involved in the regulation of feeding behavior and body weight [70,71].

In support of this evidence, Smith et al. conducted a series of experiments in male rats treated with NPS, comparing their responses in eating behavior, locomotor activity, and alteration of several hormones, including NPY. In accordance with Beck et al. results, no change in the release of NPY was revealed in in vitro study from hypothalamic explants treated with NPS. On the other hand, a significant increment of CRF and arginine vasopressin (AVP) release was found, suggesting the stimulation of hypothalamic-pituitary-adrenal (HPA) axis.

Indeed, in ad libitum fed rats, centrally injected with NPS compared to saline, significantly high levels of adrenocorticotropic hormone (ACTH) and corticosterone were detected in plasma, without significant differences in TSH or LH release. Thus, the outcomes of this neuropeptide on the HPA axis emphasize the possible mediation via the hypothalamus [18].

For what concerns locomotion and eating behavior, it was proved that ICV administration of NPS promoted rearing activity, hyperlocomotion and horizontal movements, with a significant reduction in sleeping compared with saline-treated animals. Similarly, PVN injection induced a significant increase in rearing activity and a decrease in grooming activity compared with control rats. Hence, NPS was also investigated on food intake, showing a reduction in feeding with 10 and 30 nmol of NPS (ICV) in 24 h fasted rats and the same result was obtained through PVN administration, at doses 0.1, 0.3, or 1 nmol, that significantly decreased food consumption within the first hour after injection. No differences were found in food intake among controls and treated rats at 2, 4, and 24 h after injection [18].

This study confirmed the anorectic effects of NPS, but with a shorter period of action compared to Beck et al.’s work [61], probably due to the different strain of the rats, hours of fasting, and cannula placement, specifically in the third cerebral ventricle and PVN and not the lateral brain ventricle [18,61].

Due the effect of NPS on food intake, the interaction between NPS and CRF neurotrasmission [21] (see Section 4.1) and the potential stimulatory action on the HPA axis, as previously mentioned, Fedeli et al. decided to better explore the role of NPS and to test the selective antagonist of NPSR [D-Cys(tBu)^5^]NPS, inactive per se, in male rats with cannula placement in the PVN, lateral ventricle, lateral hypothalamus (LH), and central amygdala (CeA). These brain regions are involved in emotional response to feeding and neuroanatomical data revealed high presence of NPSR mRNA [21,72,73].

In this experiment, at first, a significant decrease in HPF consumption has been shown at both doses of NPS (0.1 or 1 nmol/rat, ICV), specifically 0.1 nmol at 30 min and 1 nmol at 15 min. Then, the co-administration of [D-Cys(tBu)^5^]NPS (20.0 or 60.0 nmol/rat) and 1 nmol of NPS reduced the NPS anorectic effect at both doses, with 60.0 nmol completely blocking the anorectic action at 30 and 60 min. Furthermore, to investigate the possible interaction between NPS and CRF system, the non-selective CRF receptors (CRFRs) antagonist alpha-helical CRF 9–41 was tested, and it was not able to counteract the NPS response on HPF intake [74].

Additionally, NPS (0.03 or 0.1 nmol/rat), directly injected into the PVN, caused a significant reduction in HPF intake at all considered time-points. Both doses had the same effect into LH only at 15 min, and on the other hand, no changes in HPF intake were observed when NPS was injected into CeA, highlighting that the PVN and LH are crucial brain areas for its hypophagic activity [74].

Conversely to previous findings, Niimi’s work reported that 1 nmol of NPS stimulated food consumption in male rats for 2 h, through its interaction with the orexin system [75], focusing on the increased c-Fos levels in the orexin-expressing neurons in the LH area [75] (for details see Section 4.2).

The same orexigenic effect was shown in Badia-Elder et al. study, where low doses of NPS (0.075 or 0.3 nmol, ICV) significantly increased food intake in alcohol-preferring and -nonpreferring rats compared to the highest dose (1.2 nmol), whereas the stimulation of appetite did not persist after 24 h. Additionally, NPS did not affect sucrose solution intake at any time point [58].

These evidences were challenged by Peng et al. work, in which ICV administrations of NPS, tested at different doses (0.001–0.1 nmol), showed once again to reduce food intake in a dose-dependent manner in fasted mice [17]. Additionally, a potent antagonist of NPSR [D-Val^5^]NPS (10 nmol) and CRFR1 antagonist NBI-27914 (2 μg) were injected, alone or in co-administration with 0.1 nmol of NPS, to confirm if the NPS actions on food intake were mediated though NPSR or CRFR1. Both these antagonists did not affect feeding per se compared to vehicle mice, but in co-administration with NPS, they showed different responses: [D-Val^5^]NPS antagonized the inhibitory action on feeding induced by NPS, meanwhile NBI-27914 did not influence the central eating-related effect, but fully antagonized the hyperlocomotion of NPS. The results suggested that the impact of NPS on food intake and locomotion acts by different neuronal networks, with the anorexigenic modulation through NPSR and not CRFR1 [17]. Another selective antagonist for NPSR, SHA 68, was tested in rats (25 or 50 mg/kg intraperitoneally (ip)) and, in line with previous evidence, NPS significantly reduced HPF intake, and SHA 68, not active per se, in combination with NPS slightly counteracted the central action of the neuropeptide, probably due to the systemic injection or alternative biological mechanisms [44].

In a following study [76], ICV administration of NPS in restricted or freely fed rats under standard chow remarkably reduced food intake at doses 1.0 and 3.0 nmol at 30 and 60 min, with no statistical difference in eating at 24 h. NPS exhibited, at 1 nmol per rat, a significant decrease in HPF intake at 15, 30, and 60 min, and only a slight reduction with lower doses (0.1 or 0.3 nmol).

Interestingly, several synthesized molecules were tested on HPF consumption: the NPSR antagonists [D-Cys(tBu)^5^]NPS and [tBu-D-Gly^5^]NPS; the partial NPSR agonists [Ala^3^]NPS and [Aib^5^]NPS [76], showing different effects on the anorexigenic action of NPS. The partial agonist [Aib^5^]NPS had the same food response as NPS, while the other compounds were able to counteract the inhibitory action only when co-administered with NPS. Specifically, the effect of NPS (1 nmol, ICV) was blocked by pre-treatment with 30 nmol of [tBu-D-Gly^5^]NPS, and with 60 nmol of both [Ala^3^]NPS and [D-Cys(tBu)^5^]NPS at all time-points. The results demonstrated the ability of the partial agonist [Ala^3^]NPS to act as an antagonist in these experimental conditions, while the compounds [tBu-D-Gly^5^]NPS and [D-Cys(tBu)^5^]NPS completely blocked NPS response in food intake; precisely the first antagonist was more powerful than the second and both can be considered as candidates for the characterization of NPS/NPSR system [76]. All the studies discussed in this section are summarized in Table 1.

### 3.2. NPS in Avian Species

In the present section, we describe the role of central NPS on food intake in chicks and quails, rather than in rodents, in support of the anorectic action of this peptide in different animal species.

Cline et al. examined in broiler-type chicks the possible NPS effect on appetite- and non-appetite-related behaviors. ICV injection of NPS at different doses (0.14, 0.28, or 0.56 nmol) showed in fasted chicks a reduction in food intake in a dose-dependent manner, from 30 to 180 min of observation post-injection, confirming previous results in rats [77]. Moreover, chicks that received injection of NPS into LH and PVN at dose 0.28 nmol, demonstrated less food consumption, indicating that both these areas are implicated in NPS modulation of feeding. Additionally, immunocytochemistry studies revealed that central NPS caused a decreased activation of c-Fos immunoreactivity in the LH, hunger-related region, and at the same time, activation of PVN, normally associated with satiety [73,78]. Furthermore, a decreased plasma corticosterone was detected, especially after 0.28 nmol injection, and in regards the behavioral response to central injection of NPS, chicks have shown reduced locomotion, exploratory, and feeding pecks and a greater tendency to deep rest [77]. To better clarify the role played by this neuropeptide, Cline et al. decided, in a subsequent study, to test NPS at the same previous ICV doses in two selected lines of chicks: low (LWS) and high (HWS) body weight, representing, respectively, avian models of hypo- and hyperphagia. Despite a similar inhibitory effect on appetite, a more pronounced and fast reduction in food consumption was found in HWS line at all doses, suggesting how the HWS line is hypersensitive even at the lowest dose of treatment with respect to the LWS line. Immunocytochemistry analysis detected, in both NPS-treated lines, a decreased c-Fos immunoreactivity in the LH, while HWS chicks had more c-Fos reactivity in the PVN, without affecting the periventricular nucleus (PHN). Conversely, LWS chicks showed completely opposite results, increasing c-Fos immunoreactivity in the PHN and not in the PVN [16]. Moreover, elevated expression levels of NPS mRNA were revealed in LWS chicks compared to HWS, and in both these lines NPS was reduced in the fasted status, denoting a role of this neuropeptides in the regulation of food intake [79]. In another avian species, the Japanese quails, hypothalamic mRNA abundance of NPS was also measured, revealing that NPS mRNA was increased in the 3-h fasted birds, with the same result in refed animals after 6-h fast, assuming that this neuropeptide plays a role in energy balance and in the appetite, especially in short-term fasting [80].

A more recent work, conducted in Japanese quails, demonstrated that ICV injection of 0.25, 0.50, or 1.00 nmol of NPS reduced both food and water intake 150 min post-injection, without showing a later compensatory mechanism [19]. Moreover, behavioral analysis revealed decreased exploratory and feeding pecks in birds injected with NPS, meanwhile the distance travelled and number of jumps were not affected by the treatment. NPS-treated quails showed increased c-Fos expression, especially in the PVN, but not in the other hypothalamic areas. In the PVN of NPS-treated animals, a greater abundance of the CRF and mesotocin (MT) (OXT homolog in avian species) mRNAs was found, but no difference in CRFRs and NPY mRNAs compared to vehicle group. Then, CRFRs antagonist astressin (6 nmol) and OXT receptor antagonist OTA (6 nmol), alone or in co-injection with NPS (0.25 nmol), were used in fasted quails to evaluate the food response: reduction in food intake was observed in birds injected with NPS alone or in co-administration with OTA, meanwhile co-injection with astressin blocked the food intake-suppressive effects of NPS, showing a food consumption similar to the vehicle group [19]. This result might be explained by a possible modulation via the CRFR pathways, in light of the attenuation of the NPS anorectic effect with the CRFRs antagonist. All the studies previously described are summarized in Table 2.

## 4. The Interaction of NPS with Other Neurotransmitters Implicated in Food Intake

### 4.1. The Anorexigenic Effect of NPS and CRF Neurotransmission

Several studies highlighted the ability of the NPS to modulate arousal and to attenuate anxiety-like behaviors. Numerous brain peptides, such as orexin A or NPY, are implicated in arousal modulation via the LC [81,82], and they are also capable to influence the activity of the HPA axis [83,84,85,86] by altering CRF neurotransmission. CRF is responsible for starting the neuroendocrine response to stress via the HPA axis and it regulates several emotional reactions at extra hypothalamic sites [87,88,89], and CRFR1 antagonist demonstrated to reduce stress-induced HPF seeking [90], withdrawal symptoms under intermittent access to HPF [91] and binge eating episode in rats [92,93,94,95,96]. Interestingly, a direct functional interaction between the NPS and CRF systems is supported by the co-expression of these neurotransmitters in the LPB of rat [21] and in the KF of mice [35] and by the enhanced neuronal activity of NPS-expressing neurons in the brainstem, after exposure to different types of stressors [35,97]. Accordingly, using the microdialysis technique, a forced swimming stress revealed to promote NPS release in the amygdala of rats [98]. Regarding the influence of NPS on HPA axis activity, in Smith et al. study, both ICV and PVN injections of NPS significantly stimulated the HPA axis, increasing plasma levels of ACTH and corticosterone, respectively at 10 and 40 min after its injections [18]. The effect of NPS on plasma corticosterone and ACTH levels appeared to be mediated by the hypothalamus, since NPS stimulates the release of CRF and AVP in in vitro studies on hypothalamic explants, while the same treatment in pituitary segments did not alter ACTH levels [18]. Behavioral analysis revealed that both ICV and PVN administrations of NPS increased rearing in rats, while only PVN injection significantly decreased food intake [18]. The PVN is a brain region strictly involved in the control of the HPA axis activity and particularly rich in CRF and AVP neurons; however, the role of the PVN is not only limited to the regulation of stress responses, but profoundly influences food intake and energy expenditure, receiving inputs from AgRP and POMC neurons of the ARC and sending dense projections to feeding-related hindbrain regions [78,99]. Noteworthy, the PVN appears one of the most important brain sites in which NPS exerts the anorectic effect, considering the lower doses required to decrease HPF intake when injected in this area compared to ICV injections [74]. However, it is not completely understood if the anorectic effect of NPS is mediated by the CRF neurotransmission, and contrasting results were obtained in different studies. Initial investigations were made by comparing the decreased food intake induced by the NPS and CRF, and it was observed that, when centrally injected at equimolar doses, both of them diminished chow intake in fasted rats, even though CRF-treated rats showed a small rebound effect in the 6 h post-injection, which did not occur with NPS administration. Additionally, NPS failed to inhibit the NPY-stimulated food intake, indicating the involvement of other neuronal pathways in contrast to the known interaction of the NPY and CRF systems [61,84].

Subsequently, in Fedeli et al.’s study, pre-treatment with the non-selective CRFRs antagonist alpha-helical CRF 9–41 did not reverse the NPS response of reducing food consumption, proposing that NPS might interact in the PVN with neuropeptidergic systems different from the CRF, at least in the regulation of food intake [74]. In line with this study, Peng et al. found that central injection of a CRFR1 antagonist was not able to counteract the NPS-induced anorectic effect, but only to significantly decrease the hyperlocomotion [17]. Therefore, from these observations it emerges that the influence of NPS on eating behavior does not involve the CRF system, which is, conversely, essential for the stimulatory effect on locomotor activity, since it is prevented by both pharmacological blockade and genetic depletion of CRFR1s [17,57,100]. On the other hand, the importance of CRF neurotransmission in mediating the anorexigenic effect of NPS was highlighted by a recent study in Japanese quails (*Coturnix Japonica*), reporting, after ICV injection of NPS, an increased expression of CRF mRNA in the PVN. Furthermore, the co-injection of the non-selective CRFRs antagonist astressin attenuated the NPS-induced decrease in food consumption, suggesting the participation of CRF system in the effect of NPS in food intake [19].

### 4.2. The Interaction between NPS and Orexin Neurotransmission

Most of the investigations regarding the influence of NPS on food intake revealed the potent anorexigenic effect of this neuropeptide, even though, as previously discussed, some studies obtained the opposite results [58,75]. In particular, Niimi found an orexigenic effect of ICV-injected NPS in Sprague-Dawley rats, although not being long lasting (observed only at 2 h time-point). Interestingly, the administration of NPS induced c-Fos immunoreactivity principally in the DMN and LH area, and additionally, double staining revealed the activation of orexin-expressing neurons in the LH area, proposing that the effect of NPS in food intake might be mediated by orexins neurotransmission [75]. Orexins/hypocretins are a peptide family, comprising the neuropeptides Orexin-A and Orexin-B, which are the endogenous ligands of two GPCRs, namely the Orexin-1 receptor (OX1R) and orexin-2 receptor (OX2R) [101]. Orexins transmission in the brain is known to promote arousal, wakefulness, appetite, and orexin-expressing neurons are predominantly located in the LH area, generally recognized as the “hunger center” [10,81,102]. The potential neuroanatomical link between the NPS and orexins system has been demonstrated by double-immunostaining for orexin-A and EGFP, in NPS/EGFP transgenic mice, in which a dense network of orexins/hypocretins fibers was detected in proximity of EGFP-positive neurons in the peri-LC and KF areas, suggesting the presence of synaptic contacts between orexins and NPS-containing neurons in the brainstem [35]. In addition, following ICV injections of NPS, c-Fos immunoreactivity was detected in the LH, perifornical area (PeF) and DMH, brain regions that are considered important sources of orexin-A projecting neurons, and a measurable expression of NPSR mRNA was detected in orexin-A positive cells of the LH [54,103,104]. From these studies, a role for the NPS as an upstream modulator of orexin transmission was postulated and the functional interaction between these two systems was found critical for the pro-arousal effect of NPS and drug-seeking behaviors [54,103,104,105]. Indeed, recently Ubaldi et al. showed that the facilitatory effect of NPS on cue-induced reinstatement of alcohol seeking was blunted when an OX1R antagonist was injected directly into the PVN and in the Bed Nucleus of the Stria Terminalis, brain regions that receive orexin-A afferents from the hypothalamus and are activated in response to NPSR stimulation [104].

From these findings, the interaction between the NPS and orexin system is possible to be hypothesized, even though it is not completely understood how this could impact the food intake. Considering the important influence of orexin-A in the regulation of feeding behavior and that OX1R antagonists represent potential pharmacological tools to treat obesity and aberrant eating patterns [10,106,107,108], future investigations should elucidate if the NPS effect on food intake is, at least, partially mediated by neuronal changes in orexin transmission.

### 4.3. The Interaction of NPS with Other Neurotransmitters and Future Perspectives

Despite most of the studies focused attention on the interaction between NPS with orexin and CRF, there are other brain systems and neurotransmitters that could be influenced by the NPS, representing potential future subjects of investigation in the context of food intake. Accordingly, a recent study performed by Grund et al. investigated whether the anxiolytic effect of the NPS involves the activation of OXT neurons in the PVN of male Wistar rats. Using a retrograde tracing approach, it was demonstrated that NPS fibers originating from the LC, innervate the PVN, where NPS is able to activate OXT neurons through the NPSRs and to promote somatodendritic OXT release, as determined respectively by Ca^2+^ imaging and microdialysis. In addition, behavioral tests (Open Field Test, Light/Dark Box and Elevated Plus Maze) revealed that pharmacological blockade as well as chemogenetic silencing of OXT neurons in the PVN, completely prevented the NPS-induced anxiolysis, revealing an essential role of OXT in the effect produced by the NPS [109]. OXT is a nonapeptide hormone produced in the supraoptic nucleus and in the PVN, implicated in multiple functions, including the modulation of stress responses [110] with the ability to attenuate anxiety-related behaviors [111,112] and to exert anorexigenic effect [113,114,115], similarly to the NPS, and the dysregulation of OXT neurotransmission could aggravate the pathophysiology of certain neuropsychiatric disorders [116,117]. Therefore, considering that NPS triggers OXT release from neurons of the PVN [109], it is not to exclude the possibility that OXT neurotransmission might participate in the inhibitory action of NPS on feeding behavior, and future studies are needed to investigate this potential interaction.

A relationship between the adenosinergic system and the NPS has been also proposed. Indeed, caffeine, a non-selective adenosine receptors antagonist is anorexigenic, stimulates locomotor activity and reduces sleep, similarly to the NPS [42,118,119] and peripheral administration of caffeine, at doses that reduce food intake and promote wakefulness, was found to alter the NPS system in the rat brain, in a time-dependent manner: in particular, acute caffeine treatment decreased NPS mRNA and increased NPSR mRNA in the brainstem without influencing the hypothalamus, while a chronic treatment promotes NPSR expression only in the hypothalamus, suggesting that the sleep- and feeding-related effects of caffeine might involve NPS neurotransmission [118]. Moreover, pre-treatment with caffeine, at a dose inactive per se, counteracted the hyperlocomotion induced by ICV injections of NPS, effect replicated by co-administration with the selective adenosine A_2A_ receptor (A_2A_AR) antagonist ZM241385, but not with the adenosine A_1_ receptor antagonist CPT [120]. This result was replicated in a later study, in which pharmacological inhibition of the ecto-5′-nucleotidase, a key enzyme in extracellular adenosine production, demonstrated to block the hyperlocomotion induced by the NPS [121], suggesting the participation of A_2A_AR in mediating the stimulatory activity of NPS. Interestingly, A_2A_AR is critically involved in drug addiction and in compulsive-like eating behaviors, considering that the A_2A_AR agonists are able to attenuate ethanol intake and to block binge-like eating for HPF in rats, in a mechanism potentially involving dopaminergic transmission in reward-related brain regions [72,122,123]. Taking into account these findings, the interconnection between the NPS and adenosine system, via A_2A_AR, should be evaluated in future studies to elucidate whether the effects of NPS on drug abuse and food intake might involve changes in adenosine neurotransmission.

Finally, the NPS was found to exert an inhibitory activity on the depolarization-evoked release of 5-hydroxytryptamine and noradrenaline in the synaptosomes isolated from the mouse prefrontal cortex (PFC), possibly through the binding of presynaptic NPSRs located on serotonergic and noradrenergic nerve terminals [124,125]. In addition, in vivo microdialysis in rats revealed that NPS dose-dependently stimulated the release of extracellular dopamine and its metabolite dihydroxyphenylacetic acid in the PFC [126], brain region in which dopaminergic transmission is critically associated with the motivational aspect of reward processes related to drugs of abuse and palatable foods [127,128,129,130,131].

The influence of the NPS on dopaminergic neurotransmission was also demonstrated by recent studies in which neuroleptics drugs, such as chlorpromazine, olanzapine, and haloperidol, significantly affect the NPS and NPSR mRNA in the rat brain, suggesting that the NPS participates in the dopamine-dependent anxiolytic activity of neuroleptics [132,133].

**Figure 2:**
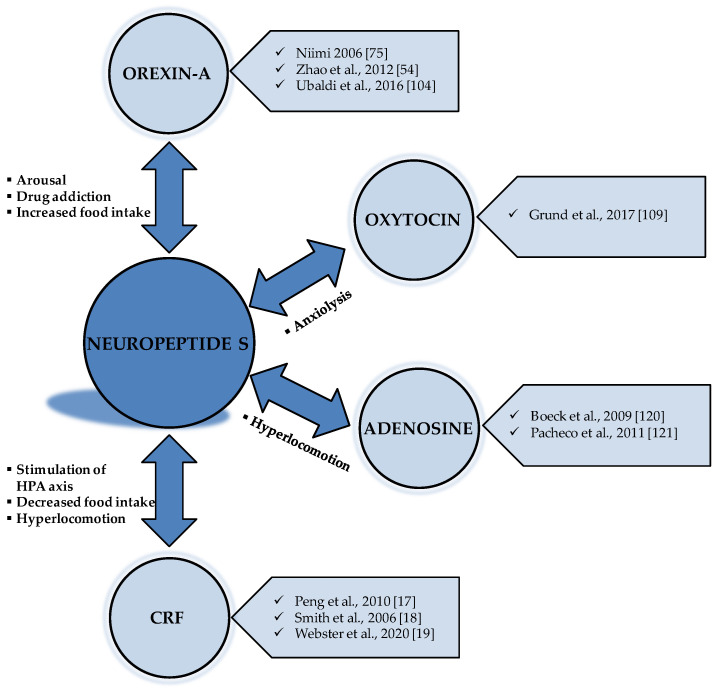
Interactions between NPS and the other neurotransmitters. CRF: corticotropin-releasing factor; HPA axis: hypothalamic-pituitary-adrenal axis. OREXIN-A: [54,75,104]; OXYTOCIN: [109]; ADENOSINE: [120,121]; CRF: [17,18,19].

The principal interconnections between NPS and the other neurotransmitters and their related effects are shown in Figure 2.

## 5. The Gastrointestinal Functions Influenced by NPS

The first study concerning the NPS system on the gastrointestinal apparatus was directed to the evaluation of this neuropeptide action on the distal colon in rodents, specifically focusing on fecal pellet output and bead expulsion [134], considered as a colonic transit tests [135,136,137]. Even though low levels of NPSR mRNA were detected in gastrointestinal tract [15], Han et al. investigated how ICV or ip administration of NPS or co-injection with NPSR antagonist [D-Val^5^]NPS could affect distal colonic transit, and if the effects were mediated by the NPSR [134].

The study revealed that peripheral NPS had no effect on distal colonic motor function, meanwhile when NPS was centrally injected, both bead expulsion and fecal pellet output were significantly inhibited in mice. [D-Val^5^]NPS did not affect the colonic transit per se, but the co-administration with NPS completely reversed the inhibitory effects on motility. Moreover, in vitro study demonstrated that NPS did not influence the contractions of the distal colon, indicating only a central mechanism of the NPS/NPSR system regarding the control of visceral motor function [134]. This work had contrary results compared to those obtained by Petrella et al. that, through central injection of NPS, did not find any differences between NPS or saline treated-rats in gastrointestinal functions, specifically in the fecal pellet output, bead expulsion, gastric emptying, and gastrointestinal transit [138]. However, when the rats were exposed to restraint stress, ICV injection of NPS significantly inhibited the number and weight of fecal pellets compared to stress saline treated-rats, effect attenuated by central pre-treatment with the antagonist [D-Cys(tBu)^5^]NPS [138].

Collectively, these findings did not find a direct influence of NPS/NPSR system on basal gastrointestinal motility under physiological state, unless when stressful conditions were applied in rats, highlighting a possible role as mediator in stress-induced colonic response [138].

A recent research [139] investigated the response after ICV administration of NPS in rats exposed to acute restraint stress, finding that NPS completely restored the stress-induced uncoordinated gastrointestinal contractions and the stress-induced delayed solid gastric emptying, apparently via the orexin system.

As in rodents, biological gut functions of the NPS/NPSR system in humans are still poorly understood. Several studies explored and found in carriers of genetic alterations of the NPSR1 an increased susceptibility to inflammatory bowel diseases (IBD) and lower gastrointestinal motility measurements, leading to perturbed gut functions [140,141]. A following study, conducted by Wan Saudi and collaborators investigated the NPS/NPSR system in both rodents and humans, revealing that rats, intravenously injected with NPS, showed a significantly reduced small intestinal and colonic motility, but an increased mucosal permeability compared with control rats. Meanwhile, human immunostaining analysis revealed a strong NPS and NPSR1 immunoreactivity in gastric corpus, jejunum, ileum, and colon. Moreover, comparing healthy subjects with IBD patients, no difference in plasma NPS levels were detected. Additionally, the human gastrointestinal muscle strips, unstimulated or pre-contracted with the cholinergic agonist bethanechol, showed that NPS induced a dose-dependent relaxatory response that was more pronounced in the small intestine than in the colon [142]. Apparently, the outcomes of the study demonstrated the ability of NPS to inhibit motility in both rats and humans and, thus, the involvement in the pathogenesis of gut disorders and dysfunctions [142].

Subsequently, it was found that rats intravenously treated with NPS showed a reduction of basal duodenal bicarbonate secretion and mucosal net fluid secretion [143]. When NPS was given to rats during luminal perfusion of 15% ethanol, the neuropeptide did not affect the increase of alcohol-induced duodenal paracellular permeability, while significantly reduced, in a dose-dependent manner, the ethanol-induced increases in duodenal motility, emphasizing once again how NPS influences this factor in the gastrointestinal tract [143]. Altogether, the findings revealed that central NPS not only influences the regulation of food intake, but also the control of gastrointestinal motor function, even though a clear mechanism of action is still unknown.

## 6. Conclusions

The role of NPS in food intake, which was investigated in rodents and avian species, demonstrated the anorexigenic effect of this peptide, in both sated and fasted animal, and its ability to counteract hyperphagia, when injected centrally and in hypothalamic areas, particularly in the PVN and LH, brain areas involved in the regulation of food consumption. Few works reported that the neuropeptide, once injected, plays an appetite-stimulating action through the orexin system or, otherwise, shows a rebound effect, observed after the inhibitory outcome on food intake. Several antagonists have been tested in animals revealing that they are normally inactive per se, but able to counterbalance the effect of NPS at different dose and time-points. Moreover, partial agonists were also administered, revealing NPS-like responses or slightly antagonistic properties. Despite the mechanisms underlying the anorexigenic effect of the NPS are not completely understood, it might involve the interaction with several neurotransmitters, among which CRF and orexin-A appear as the most important, considering their critical role in the regulation of feeding behavior. However, the NPS is also able to influence the OXT, adenosine, and dopamine signaling, suggesting their potential involvement in the NPS-induced effect on appetite suppression. Future studies should investigate these interactions and highlight the role played by NPS in the gastrointestinal apparatus in light of the capacity to reduce intestine motility and the possible association with IBD.

## Figures and Tables

**Table 1:** NPS effect in food intake in rodents.

Rodents	Injection	Result	Reference
Male mice	ICV	↓ standard chow food intake in 18 h fasted mice.	[17]
Male rats	ICV	↓ standard chow food intake after an overnight fasting. ↓ HPF intake in sated rats.	[61]
Male rats	ICV	↑ 2 h standard chow food intake.	[75]
Male rats	ICV and PVN	↓ Standard chow food intake in 24 h fasted rats.	[18]
Male rats	ICV, PVN, LH and CeA	↓ HPF consumption after ICV, PVN and LH injections. No changes on HPF consumption after CeA injection.	[74]
Male rats	ICV	↓ HPF consumption.	[44]
Male rats	ICV	↓ standard chow food intake in freely feeding and 12 h food restricted rats. ↓ HPF consumption.	[76]
Female rats	ICV	↑ standard chow food intake at doses 0.075 and 0.3 nmol. No influence on sucrose solution intake.	[58]

↓: decrease; ↑: increase; CeA: central amigdala; HPF: highly palatable food; ICV: intracerebroventricular; LH: lateral hypothalamus; PVN: paraventricular nucleus of the hypothalamus.

**Table 2:** NPS effect in food intake in avian species.

Avian Species	Injection	Result	Reference
Broiler chicks	ICV, LH and PVN	↓ Food intake in 3 h faster chicks, after ICV, LH and PVN injections.	[77]
LWS and HWS chicks	ICV	↓ Food intake in both lines, but with a more pronounced effect in HWS chicks.	[16]
LWS and HWS chicks	-	↑ NPS mRNA in LWS chicks compared to HWS. ↓ NPS mRNA in LWS and HWS after 3 h of fasting.	[79]
Japanese quails	-	↑ NPS mRNA in 3 h fasted quails. ↑ NPS mRNA in refed quails, after 6 h of fasting.	[80]
Japanese quails	ICV	↓ Food intake in 6 h fasted quails.	[19]

↓: decrease; ↑: increase; HWS: high body weight; ICV: intracerebroventricular; LH: lateral hypothalamus; LWS: low body weight; NPS: neuropeptide S; PVN: paraventricular nucleus of the hypothalamus.

## Data Availability

Data sharing not applicable.
